# Incidence of and Risk factors for Mild Cognitive Impairment in Chinese Older Adults with Multimorbidity in Hong Kong

**DOI:** 10.1038/s41598-020-60901-x

**Published:** 2020-03-05

**Authors:** Zijun Xu, Dexing Zhang, Regina W. S. Sit, Carmen Wong, Jennifer Y. S. Tiu, Dicken C. C. Chan, Wen Sun, Samuel Y. S. Wong

**Affiliations:** Division of Family Medicine and Primary Health Care, JC School of Public Health and Primary Care, The Chinese University of Hong Kong, Hong Kong, China

**Keywords:** Neurological disorders, Risk factors

## Abstract

The aim of our study was to identify the incidence rate of and the risk factors for mild cognitive impairment (MCI) among Chinese older adults with multimorbidity in primary care in Hong Kong. Older adults aged 60 years and above with multimorbidity were recruited from the public primary care clinics in Hong Kong. Incidence rates were calculated with the person-years. Cox proportional hazard regression models were used to predict the risk factors for MCI. Sensitivity analysis was performed using multiple imputation. Among 462 participants included in the main analysis, 45 progressed from normal to MCI with an incidence rate of 80.9 cases per 1000 person-years. Older age (HR 2.82, 95% CI 1.26–6.28) and being single (HR 2.15, 95% CI 1.11–4.19) were risk factors for developing MCI in the multivariable regression model. A total of 660 participants were included in the sensitivity analysis, with an MCI incidence of 114.4 cases per 1000 person-years. Older age and being single remained to be risk factors for MCI among older adults with multimorbidity. There may be a high incidence of MCI among Chinese older adults with multimorbidity in primary care in Hong Kong. Future larger studies need to confirm the prevalence and incidence of MCI among primary care Chinese patients.

## Introduction

Hong Kong has one of the longest life expectancy in the world, with the average life expectancies at birth being 87.6 years for female and 82.2 years for male in 2018^1^. As lifespans expand, chronic health problems related to aging have become a public health priority and it is common for many older adults to suffer from multiple chronic diseases. Multimorbidity has been mostly used to describe the condition, defined as the coexistence of two or more chronic diseases in the same person^2^. It was reported that among the Hong Kong population aged 65 years and above, 68% had multimorbidity, with more than half of them having three or more chronic diseases^3^. Multimorbidity is associated with poorer quality of life and functional status and higher health care utilization when compared to those without^4,5^.

Mild cognitive impairment (MCI) is a common condition in older adults^6^. Although it is common among older adults, very few studies have been conducted to describe the prevalence and incidence of MCI among older adults with multiple chronic conditions in primary care settings. Previously, several longitudinal studies have been conducted to measure the relationship between multimorbidity and cognitive decline and show that multimorbidity can adversely affect cognitive function^7–9^. Furthermore, it has been shown the number of concurrent conditions is associated with an increased risk of developing MCI^10^. Older adult population who have 2 or more chronic conditions and 4 or more chronic conditions has 1.38 and 1.61 times the risk of MCI as those who only have one or no chronic condition^10^. The only study in China reported that the 5-year cumulative incidence rate of MCI was more than 10% among community-dwelling older adults aged 65 and over in Beijing^11^. So the real situation of MCI may be worse among older adults with multimorbidity in Hong Kong than that reported among community-dwelling older adults.

To slow down the cognitive decline in this high-risk population, risk factors for developing MCI need to be recognized. Many modifiable factors have been shown to be related to the risk of MCI among healthy older adults^12^. Recent systematic reviews and meta-analysis have suggested that risk factors of vascular diseases can play a role in the cognitive decline and development of MCI which include high total serum cholesterol^13^, alcohol consumption^14^, a low level of physical activity^15^, diabetes^16^, insomnia^17^, loneliness^18^ and the presence of neuropsychiatric symptoms^19^.

Only two studies in Asia have measured the MCI incidence and risk factors among older adults^11,20^. We therefore used data from a primary care community cohort to examine the incidence of and the risk factors for MCI among Chinese older adults with multimorbidity in primary care in Hong Kong such that further care for older adults in the management and prevention of cognitive impairment can be planned.

## Methods

### Study design

This is a longitudinal study reporting the MCI incidence and risk factors in a primary care cohort for multimorbidity in Hong Kong. The study was approved by the Joint Chinese University of Hong Kong - New Territories East Cluster Clinical Research Ethics Committee (CREC2016.204). All research was performed in accordance with relevant guidelines and regulations. Informed consent was obtained from all participants.

### Study setting and participants

Public primary care patients were recruited from four general out-patient clinics (GOPCs) in the New Territory East Cluster (NTEC) of Hong Kong. The population of NTEC was estimated to be approximately 800,000 in 2018^21^. According to the Hong Kong Hospital Authority Annual Report 2017–2018, a total of 983,997 consultations were provided by ten GOPCs in NTEC in a year^22^.

The inclusion criteria for the cohort were: 1) aged 60 years old or above; 2) suffering from two or more chronic diseases confirmed by the healthcare information in the public Clinical Management System (CMS) and participants’ self-report; 3) could speak Cantonese and understand Chinese; 4) could come to the clinic to sign informed consent by themselves, and being able to understand and respond to assessment consisting of questionnaire survey conducted by trained staff.

Patients who met the inclusion criteria were recruited to the cohort. After recruitment and baseline assessments, activities targeting mental, physical and social health were provided to the patients according to their personal preferences which include exercise and nutritional classes, counseling, and health talks. About 40% of the participants joined the activities. Trained health professionals conducted the baseline and follow-up assessments through face-to-face interviews at a university-affiliated primary care clinic. Participants with normal cognition at baseline were included in this study.

### Measurements

MCI was measured by Hong Kong Montreal Cognitive Assessment (HK-MoCA) both at baseline and follow-up^23^. After adjusting for years of education (+1 point if year of education no more than 6), a total score of less than 22 was identified as MCI. The cutoff value of 21/22 has been validated locally among older adults^24^.

Potential risk factors of MCI were measured at baseline. Social demographic information including age, gender, years of education, marriage status, living alone, working status, social assistance, comprehensive social security assistance (CSSA) scheme were reported. A body mass index (BMI) within the range of 18.5–23.9 was considered normal according to the Chinese standard^25^. Clinically relevant depressive symptoms were measured by the 9-item Patient Health Questionnaire (PHQ-9) with a score of 5 or more suggesting possible depression^26^. Anxiety was measured using 7-item Generalised Anxiety Disorder (GAD-7), with a score of 5 or more suggesting anxiety^27^. Loneliness was measured by the 6-item De Jong Gierveld Loneliness Scale^28^. Insomnia was measured by the 7-item Insomnia Severity Index (ISI) among those who had an affirmative answer to a screening question “Did you have insomnia in the past two weeks”^29^. Frailty was measured by the Edmonton Frail Scale and was classified into no frailty, pre-frailty and frailty^30^. Sarcopenia was measured by the 5-item Sarcopenia Assessment (SARC-F)^31^. Alcohol use was measured by the 3-item Alcohol Use Disorders Identification Test-consumption (AUDIT-C)^32^. Smoking status, social media use, incontinence, oral health problem, social support, and perceived health were also measured. Blood pressure was measured twice by nurses using standard method^33^. Past medical history was documented using data from the public CMS which included doctor-diagnosed diseases and long-term use of medications.

### Data analysis

Participants with normal cognition at baseline and with HK-MOCA score available at first follow up assessment were included in the main analysis. Baseline characteristics were presented as numbers and percentages. Chi-square tests were used to assess the differences in baseline characteristics between different age groups. Chi-square tests were also used to assess the differences between the study population and participants lost to follow-up, as well as between the study population and patients with three or more chronic conditions from GOPCs from another study, which can represent the general primary care patients with multimorbidity in Hong Kong^34^. The incidence of MCI for the study population and for participants with different potential risk factors were calculated as the number of cases per 1000 person-years at risk and their 95% confidence intervals (CIs). Person-years were calculated by the time from baseline to the first follow-up assessment. Cox proportional hazard regression models were used to analyze the effect of baseline characteristics as risk factors on incident MCI over time. The results of the model were presented as hazard ratios (HRs) and their 95% CIs. Factors with a significant level less than 0.1 in the univariable regression model were entered in multivariable Cox proportional hazard model for adjustment. Sensitivity analysis was performed by imputing missing data for those did not complete the follow-up assessment. Follow-up durations were imputed using the average follow-up year. Missing follow-up HK-MoCA scores were accounted for using age, gender, years of education, social assistance, and social media use by multiple imputation with chained equations^35^. Ten imputed data sets were created and analyzed together to get a pooled incidence and pooled HRs both in univariable and multivariable regression. All analyses were conducted using Stata version 13.1. A p-value of less than 0.05 (two sides) was considered statistically significant.

## Results

**Figure 1:**
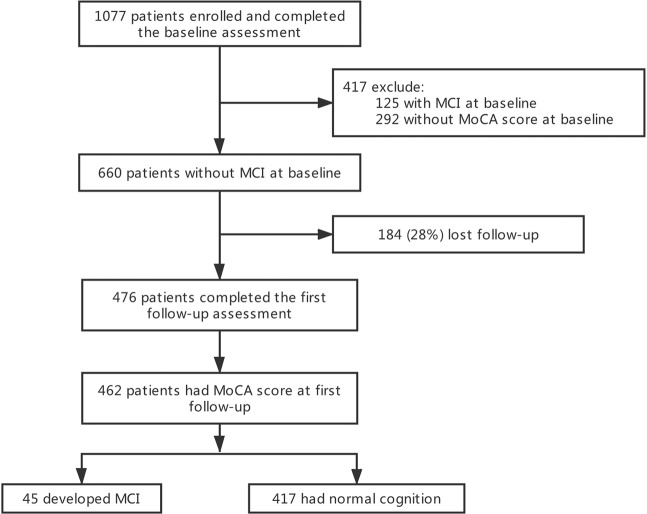
Flowchart of the study. MCI: mild cognitive impairment; MoCA: Montreal Cognitive Assessment.

A total of 1077 patients who met the inclusion criteria were recruited and completed the baseline assessments from April 2016 to October 2017 (Fig. 1). Among them, 417 were excluded from this study due to MCI (n = 125) and unavailable HK-MoCA score (n = 292) at baseline. Out of the 660 participants with normal cognition at baseline, 184 (28%) were lost follow-up, and 476 (72%) completed the first follow-up assessments from April 2018 to March 2019, with an average follow-up 1.4 years (range: 0.8–2.7 years). A total of 462 patients had HK-MoCA score at first follow-up assessment and were included in the main analysis.

Demographic characteristics are summarized in Table 1. Among 462 participants, 145 (31.4%) were male; 284 (61.6%) had BMI lower than 18.5 kg/m^2^ or more than 23.9 kg/m^2^; 204 (44.2%) had fewer than 6 years of education; 145 (31.4%) were single, divorced, separated or widowed; 64 (13.9%) lived alone; 416 (90.0%) were unemployed; 226 (48.9%) had social assistance and 29 (6.3%) had CSSA. Gender, year of education, marriage status, working status, and social assistance were significantly different between participants aged 60 to 69 years, 70 to 79 years and over 80 years (P values < 0.05). The 462 participants had an average MoCA score of 26.2 (SD = 0.1, median = 25) at baseline.

**Table 1:** Demographic characteristics of the 462 participants included in the main analysis.

Demographic characteristics	Age				Study sample (n = 462)
	60–69 (n = 281)	70–79 (n = 154)	80+(n = 26)	P	
Gender				0.020	
Male	75 (16.2)	61 (13.2)	9 (1.9)		145 (31.4)
Female	206 (44.6)	93 (20.1)	17 (3.7)		317 (68.6)
BMI				0.726	
Normal BMI	104 (22.5)	63 (13.6)	10 (2.2)		177 (38.4)
Abnormal BMI	177 (38.3)	91 (19.7)	16 (3.5)		284 (61.6)
Education				< 0.001	
> 6y	173 (37.4)	80 (17.3)	5 (1.1)		258 (55.8)
<= 6y	108 (23.4)	74 (16)	21 (4.5)		204 (44.2)
Marriage				< 0.001	
Married	202 (43.7)	105 (22.7)	9 (1.9)		317 (68.6)
Single/divorced/separated/widowed	79 (17.1)	49 (10.6)	17 (3.7)		145 (31.4)
Live alone				0.076	
No	248 (53.7)	130 (28.1)	19 (4.1)		398 (86.1)
Yes	33 (7.1)	24 (5.2)	7 (1.5)		64 (13.9)
Working status				0.002	
Unemployed	242 (52.4)	148 (32)	25 (5.4)		416 (90.0)
Employed	39 (8.4)	6 (1.3)	1 (0.2)		46 (10.0)
Social assistance				< 0.001	
No	216 (46.8)	16 (3.5)	3 (0.6)		236 (51.1)
Yes	65 (14.1)	138 (29.9)	23 (5)		226 (48.9)
CSSA				0.209	
No	267 (57.8)	140 (30.3)	25 (5.4)		433 (93.7)
Yes	14 (3)	14 (3)	1 (0.2)		29 (6.3)

BMI: body mass index; CSSA: comprehensive social security assistance.

In Table 2, the demographic characteristics of 462 participants were compared with those of 530 GOPC patients with three or more chronic conditions in another study in Hong Kong. Age, gender, year of education, social assistance, and CSSA were significantly different between the two groups (P values < 0.05). The demographic characteristics were also compared with 184 participants lost to follow-up and no significant difference was found (P values > 0.05).

**Table 2:** Demographic characteristics of the study sample compared with 530 patients with three or more chronic conditions from GOPCs.

Demographic characteristics	Study sample (n = 462)	Patients from GOPCs (n = 530)	P*	Lost to follow-up (n = 184)	P#
Age			< 0.001		0.199
60–69	281 (61.0)	194 (36.6)		113 (61.4)	
70–79	154 (33.4)	212 (40.0)		54 (29.4)	
80+	26 (5.6)	124 (23.4)		17 (9.2)	
Gender			< 0.001		0.273
Male	145 (31.4)	296 (56.0)		66 (35.9)	
Female	317 (68.6)	233 (44.0)		118 (64.1)	
Education			0.005		0.925
> 6y	258 (55.8)	247 (46.9)		102 (55.4)	
< = 6y	204 (44.2)	280 (53.1)		82 (44.6)	
Marriage			0.167		0.234
Married	317 (68.6)	382 (72.6)		135 (73.4)	
Single/divorced/separated/widowed	145 (31.4)	144 (27.4)		49 (26.6)	
Live alone			0.619		0.072
No	398 (86.1)	443 (85.0)		168 (91.3)	
Yes	64 (13.9)	78 (15.0)		16 (8.7)	
Working status			0.955		0.345
Unemployed	416 (90.0)	476 (90.2)		161 (87.5)	
Employed	46 (10.0)	52 (9.8)		23 (12.5)	
Social assistance			< 0.001		0.617
No	236 (51.1)	124 (23.4)		98 (53.3)	
Yes	226 (48.9)	406 (76.6)		86 (46.7)	
CSSA			< 0.001		0.126
No	433 (93.7)	460 (86.8)		178 (96.7)	
Yes	29 (6.3)	70 (13.2)		6 (3.3)	

^*^Comparing patients from GOPCs with the study sample. ^#^Comparing patients lost to follow-up with the study sample. CSSA: comprehensive social security assistance; GOPC: general out-patient clinic.

Among 462 participants, 45 progressed from normal to MCI, with an incidence rate of 80.9 cases per 1000 person-years (95% CI 60.4–108.3 per 1000 person-years). The incidence rates of each subgroup, crude and adjusted HR (95% CI) were summarized in Supplementary Table S1 online. Variables with a P value of less than 0.1 in univariable regression were shown in Table 3. The Cox regression showed that significantly increased risk of MCI was associated with older age (70–79 compared with 60–69 years old: HR 4.03, 95% CI 2.05–7.92; 80+ compared with 60–69 years old: HR 6.24, 95% CI 2.45–15.84), less education (≤6 years compared with > 6 years: HR 2.37, 95% CI 1.29–4.34), unmarried status (single/divorced/separated/widowed compared with married: HR 2.62, 95% CI 1.46–4.70), having social assistance (HR 3.07, 95% CI 1.59–5.95), incontinence (HR 2.10, 95% CI 1.12–3.91), regular medication use (≥5 compared with <5: HR 2.06, 95% CI 1.13–3.74), and antidiabetic use (HR 2.05, 95% CI 1.12–3.75). Social media use (HR 0.32, 95% CI 0.18–0.58) was associated with a decreased risk of MCI.

**Table 3:** Factors associated with the risk of MCI among 462 cognitively normal older adults with multimorbidity.

Variable	Crude HR (95% CI)	P Value	Multivariable-Adjusted HR (95% CI)	P Value
Age				
60–69	1 (Reference)	NA	1 (Reference)	NA
70–79	4.03 (2.05, 7.92)	< 0.001	2.55 (1.08, 6.01)	0.032
80+	6.24 (2.45, 15.84)	< 0.001	2.05 (0.67, 6.22)	0.206
Education				
> 6y	1 (Reference)	NA	1 (Reference)	NA
<= 6y	2.37 (1.29, 4.34)	0.005	1.35 (0.69, 2.65)	0.380
Marriage				
Married	1 (Reference)	NA	1 (Reference)	NA
Single/divorced/separated/widowed	2.62 (1.46, 4.70)	0.001	1.99 (1.01, 3.92)	0.048
Social assistance				
No	1 (Reference)	NA	1 (Reference)	NA
Yes	3.07 (1.59, 5.95)	0.001	1.24 (0.53, 2.91)	0.619
Social media use				
No	1 (Reference)	NA	1 (Reference)	NA
Yes	0.32 (0.18, 0.58)	< 0.001	0.53 (0.27, 1.04)	0.064
Loneliness				
Total score = 0	1 (Reference)	NA	1 (Reference)	NA
Total score >= 1	0.56 (0.31, 1.01)	0.054	0.56 (0.30, 1.07)	0.079
Sarcopenia				
No	1 (Reference)	NA	1 (Reference)	NA
Yes	2.11 (0.89, 5.02)	0.090	0.97 (0.32, 2.94)	0.953
Frailty				
No frailty	1 (Reference)	NA	1 (Reference)	NA
Pre-frailty	1.10 (0.57, 2.12)	0.787	1.17 (0.57, 2.38)	0.664
Frailty	2.08 (0.91, 4.73)	0.081	0.97 (0.33, 2.86)	0.963
Incontinence				
No	1 (Reference)	NA	1 (Reference)	NA
Yes	2.10 (1.12, 3.91)	0.020	1.21 (0.58, 2.52)	0.618
Hypertension (diagnosed)				
No	1 (Reference)	NA	1 (Reference)	NA
Yes	1.99 (0.92, 4.26)	0.079	1.24 (0.54, 2.84)	0.612
Diabetes mellitus (diagnosed)				
No	1 (Reference)	NA	1 (Reference)	NA
Yes	1.76 (0.97, 3.20)	0.064	0.92 (0.24, 3.61)	0.909
Regular medication use				
< 5	1 (Reference)	NA	1 (Reference)	NA
> = 5	2.06 (1.13, 3.74)	0.018	0.95 (0.41, 2.15)	0.895
Antidiabetics use				
No	1 (Reference)	NA	1 (Reference)	NA
Yes	2.05 (1.12, 3.75)	0.020	2.04 (0.50, 8.31)	0.319
Analgesics use				
No	1 (Reference)	NA	1 (Reference)	NA
Yes	2.04 (0.98, 4.25)	0.055	1.40 (0.55, 3.53)	0.479

CI: confidence interval; HR: hazard ratio; MCI: mild cognitive impairment; NA: not available.

In multivariable Cox regression, older age (70–79 compared with 60–69 years old: HR 2.55, 95% CI 1.08–6.01) and unmarried status (single/divorced/separated/widowed compared with married: HR 1.99, 95% CI 1.01–3.92) continued to be associated with increased risk of developing MCI (Table 3). Year of education, social assistance, social media use, incontinence, regular medication use, and antidiabetics use were no longer significantly associated with incidence MCI after adjustment (P values > 0.05). Loneliness, sarcopenia, frailty, diagnosed hypertension, diagnosed diabetes mellitus, and analgesics use were included in the multivariable regression model but continued to be non-significant (P values > 0.05).

After running the multiple imputation to impute the missing data, a total of 660 participants were included in the sensitivity analysis. The pooled incidence of MCI was 114.4 cases per 1000 person-years (95% CI 107.4–121.9 per 1000 person-years) using the imputed data sets.

Supplementary Table S2 online summarized the factors associated with the risk of MCI among 660 participants after multiple imputation. Variables with a P value of less than 0.1 in univariable regression were shown in Table 4. Older age (70–79 compared with 60–69 years old: HR 4.00, 95% CI 2.04–7.83; 80+ compared with 60–69 years old: HR 4.72, 95% CI 1.88–11.87), less education (HR 2.28, 95% CI 1.24–4.18), unmarried status (HR 2.81, 95% CI 1.56–5.04), having social assistance (HR 3.05, 95% CI 1.57–5.91), incontinence (HR 2.02, 95% CI 1.09–3.76), regular medication use (HR 1.85, 95% CI 1.02–3.35) continued to associate with increased risk of MCI. Social media use (HR 0.40, 95% CI 0.22–0.73) was still associated with a decreased risk of MCI. Antidiabetic use (HR 1.81, 95% CI 0.99–3.30) was not significantly associated with MCI after multiple imputation.

**Table 4:** Factors associated with the risk of MCI among 660 participants after multiple imputation.

Variable	Crude HR (95% CI)	P Value	Multivariable-Adjusted HR (95% CI)	P Value
Age				
60–69	1 (Reference)	NA	1 (Reference)	NA
70–79	4.00 (2.04, 7.83)	< 0.001	2.99 (1.21, 7.36)	0.017
80+	4.72 (1.88, 11.87)	0.001	2.31 (0.75, 7.13)	0.145
Education				
> 6y	1 (Reference)	NA	1 (Reference)	NA
< = 6y	2.28 (1.24, 4.18)	0.007	1.69 (0.89, 3.20)	0.110
Marriage				
Married	1 (Reference)	NA	1 (Reference)	NA
Single/divorced/separated/widowed	2.81 (1.56, 5.04)	0.001	2.06 (1.10, 3.88)	0.025
Social assistance				
No	1 (Reference)	NA	1 (Reference)	NA
Yes	3.05 (1.57, 5.91)	0.001	1.06 (0.43, 2.59)	0.905
Social media use				
No	1 (Reference)	NA	1 (Reference)	NA
Yes	0.40 (0.22, 0.73)	0.003	0.71 (0.37, 1.39)	0.318
Frailty				
No frailty	1 (Reference)	NA	1 (Reference)	NA
Pre-frailty	1.10 (0.57, 2.12)	0.784	1.11 (0.56, 2.22)	0.759
Frailty	2.19 (0.96, 4.98)	0.061	1.21 (0.47, 3.09)	0.690
Incontinence				
No	1 (Reference)	NA	1 (Reference)	NA
Yes	2.02 (1.09, 3.76)	0.026	1.32 (0.68, 2.59)	0.412
Regular medication use				
< 5	1 (Reference)	NA	1 (Reference)	NA
> = 5	1.85 (1.02, 3.35)	0.044	0.89 (0.39, 2.00)	0.755
Antidiabetics use				
No	1 (Reference)	NA	1 (Reference)	NA
Yes	1.81 (0.99, 3.30)	0.054	1.76 (0.85, 3.64)	0.125
Analgesics use				
No	1 (Reference)	NA	1 (Reference)	NA
Yes	1.93 (0.93, 4.00)	0.078	1.61 (0.68, 3.85)	0.280

CI: confidence interval; HR: hazard ratio; MCI: mild cognitive impairment; NA: not available.

In multivariable Cox regression after multiple imputation, older age (70–79 compared with 60–69 years old: HR 2.99, 95% CI 1.21–7.36) and unmarried status (single/divorced/separated/widowed compared with married: HR 2.06, 95% CI 1.10–3.88) were associated with increased risk of developing MCI among older adults with multimorbidity (Table 4).

## Discussion

This longitudinal analysis of a Chinese primary care cohort of older adults aged 60 years and older with multimorbidity in Hong Kong shows that the incidence of MCI was estimated to be 80.9 per 1000 person-years. Older age and non-married status were associated with a 2 to 3 time higher risk of developing MCI.

A systematic review conducted in 2008 showed that the overall incidence rate of MCI among people aged 65 or above ranged from 21.5 to 71.3 per 1000 person-years^36^. In another systematic review, the overall incidence rate of MCI ranged between 8.5 and 76.8 per 1000 person-years among population- or community-based samples in western countries^37^. There were only two studies which measured MCI incidence in Asia, not included in the two systematic reviews above. Bae’s study in Korea showed the incidence rate was 28.1 per 1000 person-years among general population aged 65 years and above^20^. Zhuo’s study in China reported that an average annual incidence rate of MCI was 2.17% among community-dwelling older adults aged 65 and over in a five-year follow-up study in Beijing^11^. Another study in the United States in clinic-based samples indicated an overall incidence of MCI as 77.3 cases per 1000 person-years^38^. The current study reports a higher incidence of cognitive impairment when compared to findings from the systematic reviews and other studies. This may be due to the presence of multiple chronic conditions of primary care patients in our study which have put the participants at higher risk of developing MCI. A study has found the risk of MCI was higher in older adults with multimorbidity than in those with less than two chronic conditions^10^. Potential mechanisms are aging, multiple chronic conditions and cognitive impairment both having the same etiologies or through cardiovascular diseases, or the individual or combined effects of two or more chronic conditions leading to pathological change^10^. However, the incidence of MCI may also be dependent on the different diagnostic criteria applied, different tools used and different cutoff values being used for various diagnostic tools^36,39^. For example, Petersen’s standard^40^, MoCA^24^, Mini-Mental State Examination^41^ were all widely used to define MCI. We have chosen using the MoCA in our current study as it has been validated locally and recommended by the government to screen MCI^24,42^.

In our study, older age was found to be a risk factor for MCI, with people aged 70–79 having almost three times the risk of developing MCI when compared to those aged 60–69. This is consistent with findings from previous studies^43^. With increasing age, the brain tissue of the older adults begin to shrink, and physiological function gradually declines^44^. We also found that unmarried people had two times the risk of developing MCI when compared to those who are married. Bae’s study reported that although non-married (single, widowed, or divorced) was a risk factor for Alzheimer’s disease with an HR more than 8, it was not a significant risk factor for MCI^20^. Unmarried people may have a different lifestyle when compared to those of married people. For example, they may not need to take care of their partners. A study also found that living without partners may increase the risk of smoking and alcohol consumption, having irregular breakfast and prevent them from undergoing health screening^45^.

Having less education was found to increase the risk of developing MCI before adjustment in our study. Whether education is a risk factor for MCI is uncertain. Both Solfrizzi and Tervo’s study found healthy older adults with higher education level were less likely to develop MCI than those with lower or no education^46,47^. Manly’s study found that education was not a significant risk factor for MCI^43^. Social media use was also related to the decreasing risk of MCI before adjustment. Randomized controlled trials have demonstrated social media can play a beneficial role in enhancing cognitive health among older adults^48^. Incontinence was associated with the increasing risk of developing MCI in our study. A study reported that dementia would contribute to incontinence among elderly patients^49^. Future research should further study the bi-directional relationship between incontinence and MCI since very few studies have been conducted in this area. Regular medication use and antidiabetics use were associated with increased risk of MCI before adjustment. Meta-analysis has shown that people with diabetes had 20% more risk of developing MCI than those without diabetes^16^. However, in our study, doctor-diagnosed diabetes mellitus was not a risk factor for MCI, which may have been due to the limited sample size. In addition, the effect of drug use may have been confounded by age. As age increases, the prevalence of chronic conditions and multimorbidity increases and more medications are needed. However, these factors were no longer significantly associated with incident MCI after adjustment.

This study had a dropout rate of almost 30%. Although there was no significant difference between the the study sample and those lost to follow-up, sensitivity analysis was performed to account for missing data using multiple imputation. After multiple imputation, the incidence of MCI increased to 114.4 cases per 1000 person-years, which was higher than the original incidence and those of previous studies. Participants who did not return for follow-up may have suffered from worsening physical and mental condition including MCI. The real situation of MCI in Hong Kong may be underestimated. In terms of risk factors for developing MCI, older age and being single were associated with increased risk of MCI after the multiple imputation procedure, consistent with the results from the main data analysis.

This study also had several limitations. First, the participants were self-selected ambulatory sample, and was younger, more educated, more female, and having less social assistance and CSSA than the general patients with multiple chronic conditions from GOPCs without excluding those with MCI. It may have resulted in selecting the healthier or higher functioning primary care patients with multimorbidity. Second, some participants received interventions in the programme according to their personal preference, which may have influenced the incidence of MCI, leading to a lower incidence. The third limitation is the high level of loss to follow-up. Although these missing data were addressed through multiple imputation, our data are still subjected to selection bias due to loss to follow-up. Moreover, we only measured the MCI incidence and risk factors among older adults with multimorbidity. Future studies should also investigate the incidence rate of MCI in general older adults in Hong Kong.

## Conclusions

Our findings showed that there may be a high incidence of MCI among Chinese primary care patients with multiple chronic conditions in Hong Kong. Future larger studies are needed to further confirm the prevalence and incidence of MCI among primary care Chinese patients such that evidence-based interventions can be made available for this emerging public health issue.

## Supplementary information

Supplementary Information.

## Data Availability

The datasets generated during and/or analysed during the current study are available from the corresponding author on reasonable request.
