# Supplementary material for: Incidence of and Risk factors for Mild Cognitive Impairment in Chinese Older Adults with Multimorbidity in Hong Kong
Source: Sci Rep. 2020 Mar 5;10:4137. doi: 10.1038/s41598-020-60901-x (PMC7057945; doi:10.1038/s41598-020-60901-x)
Supplement: Supplementary file 1 — Supplementary Information. [file 41598_2020_60901_MOESM1_ESM.docx]

**Supplementary Information**

**Incidence of and Risk factors for Mild Cognitive Impairment in Chinese Older Adults with Multimorbidity in Hong Kong**

Zijun XU, Dexing ZHANG, Regina WS SIT, Carmen WONG, Jennifer YS TIU, Dicken CC Chan, Wen Sun, Samuel YS WONG

Supplementary Table S1 Incidence and factors associated with the risk of MCI among 462 cognitively normal older adults with multimorbidity

| Variable | Incident MCI  Cases, No. | Person-years at Risk | Cases per 1000  Person-years (95%CI) | Crude HR  (95%CI) | P Value | Multivariable-Adjusted HR (95%CI) | P Value |
| --- | --- | --- | --- | --- | --- | --- | --- |
| Total | 45 | 556.4 | 80.9 (60.4, 108.3) |  |  |  |  |
| Age |  |  |  |  |  |  |  |
| 60-69 | 13 | 341.6 | 38.1 (22.1, 65.5) | 1 (Reference) | NA | 1 (Reference) | NA |
| 70-79 | 25 | 182.4 | 137.1 (92.6, 202.8) | 4.03 (2.05, 7.92) | <0.001 | 2.55 (1.08, 6.01) | 0.032 |
| 80+ | 7 | 31.6 | 221.6 (105.7, 464.9) | 6.24 (2.45, 15.84) | <0.001 | 2.05 (0.67, 6.22) | 0.206 |
| Gender |  |  |  |  |  |  |  |
| Male | 12 | 172.0 | 69.8 (39.6, 122.8) | 1 (Reference) | NA |  |  |
| Female | 33 | 384.4 | 85.8 (61.0, 120.8) | 1.14 (0.59, 2.20) | 0.703 |  |  |
| BMI |  |  |  |  |  |  |  |
| Normal BMI | 18 | 216.0 | 83.3 (52.5, 132.3) | 1 (Reference) | NA |  |  |
| Abnormal BMI | 27 | 340.4 | 79.3 (54.4, 115.7) | 0.97 (0.53, 1.75) | 0.908 |  |  |
| Education |  |  |  |  |  |  |  |
| >6y | 17 | 315.1 | 54.0 (33.5, 86.8) | 1 (Reference) | NA | 1 (Reference) | NA |
| <=6y | 28 | 241.3 | 116.0 (80.1, 168.1) | 2.37 (1.29, 4.34) | 0.005 | 1.35 (0.69, 2.65) | 0.38 |
| Marriage |  |  |  |  |  |  |  |
| Married | 21 | 382.6 | 54.9 (35.8, 84.2) | 1 (Reference) | NA | 1 (Reference) | NA |
| Single/divorced/  separated/widowed | 24 | 173.8 | 138.1 (92.6, 206.0) | 2.62 (1.46, 4.70) | 0.001 | 1.99 (1.01, 3.92) | 0.048 |
| Live alone |  |  |  |  |  |  |  |
| No | 37 | 480.1 | 77.1 (55.8, 106.2) | 1 (Reference) | NA |  |  |
| Yes | 8 | 76.3 | 104.8 (52.4, 209.5) | 1.50 (0.70, 3.22) | 0.303 |  |  |
| Working status |  |  |  |  |  |  |  |
| Unemployed | 41 | 500.7 | 81.9 (60.3, 111.2) | 1 (Reference) | NA |  |  |
| Employed | 4 | 55.7 | 71.8 (27.0, 191.3) | 0.85 (0.30, 2.37) | 0.752 |  |  |
| Social assistance |  |  |  |  |  |  |  |
| No | 12 | 287.0 | 41.8 (23.7, 73.6) | 1 (Reference) | NA | 1 (Reference) | NA |
| Yes | 33 | 269.4 | 122.5 (87.1, 172.3) | 3.07 (1.59, 5.95) | 0.001 | 1.24 (0.53, 2.91) | 0.619 |
| CSSA |  |  |  |  |  |  |  |
| No | 40 | 520.7 | 76.8 (56.4, 104.8) | 1 (Reference) | NA |  |  |
| Yes | 5 | 35.8 | 139.8 (58.2, 335.8) | 1.62 (0.63, 4.14) | 0.314 |  |  |
| Social media use |  |  |  |  |  |  |  |
| No | 27 | 170.0 | 158.8 (108.9, 231.6) | 1 (Reference) | NA | 1 (Reference) | NA |
| Yes | 18 | 386.4 | 46.6 (29.3, 73.9) | 0.32 (0.18, 0.58) | <0.001 | 0.53 (0.27, 1.04) | 0.064 |
| Depression (PHQ9) |  |  |  |  |  |  |  |
| No | 39 | 451.4 | 86.4 (63.1, 118.3) | 1 (Reference) | NA |  |  |
| Yes | 6 | 105.1 | 57.1 (0.26, 127.1) | 0.55 (0.23, 1.32) | 0.181 |  |  |
| Anxiety (GAD7) |  |  |  |  |  |  |  |
| No | 40 | 451.5 | 88.6 (65.0, 120.8) | 1 (Reference) | NA |  |  |
| Yes | 5 | 105.0 | 47.6 (19.8, 114.4) | 0.47 (0.18, 1.19) | 0.111 |  |  |
| Loneliness |  |  |  |  |  |  |  |
| Total score =0 | 21 | 190.1 | 110.5 (72.0, 169.5) | 1 (Reference) | NA | 1 (Reference) | NA |
| Total score >=1 | 24 | 329.4 | 72.9 (48.8, 108.7) | 0.56 (0.31, 1.01) | 0.054 | 0.56 (0.30, 1.07) | 0.079 |
| Insomnia |  |  |  |  |  |  |  |
| No | 20 | 258.8 | 77.3 (49.9, 119.8) | 1 (Reference) | NA |  |  |
| Yes | 25 | 297.6 | 84.0 (56.8, 124.3) | 1.44 (0.80, 2.60) | 0.223 |  |  |
| Sarcopenia |  |  |  |  |  |  |  |
| No | 39 | 482.8 | 80.8 (59.0, 110.6) | 1 (Reference) | NA | 1 (Reference) | NA |
| Yes | 6 | 29.9 | 201.0 (90.3, 447.3) | 2.11 (0.89, 5.02) | 0.09 | 0.97 (0.32, 2.94) | 0.953 |
| Drinker |  |  |  |  |  |  |  |
| No | 42 | 527.2 | 79.7 (58.9, 107.8) | 1 (Reference) | NA |  |  |
| Yes | 3 | 29.2 | 102.7 (33.1, 318.4) | 1.47 (0.45, 4.77) | 0.518 |  |  |
| Smoker |  |  |  |  |  |  |  |
| Non-smoker | 39 | 474.9 | 82.1 (60.0, 112.4) | 1 (Reference) | NA |  |  |
| Current smoker | 2 | 18.2 | 110.2 (27.5, 440.4) | 1.33 (0.32, 5.50) | 0.696 |  |  |
| Ex-smoker | 4 | 63.4 | 63.1 (23.7, 168.0) | 0.85 (0.30, 2.38) | 0.759 |  |  |
| Frailty |  |  |  |  |  |  |  |
| No frailty | 16 | 233.4 | 68.5 (42.0, 111.9) | 1 (Reference) | NA | 1 (Reference) | NA |
| Pre-frailty | 20 | 270.0 | 74.1 (47.8, 114.8) | 1.10 (0.57, 2.12) | 0.787 | 1.17 (0.57, 2.38) | 0.664 |
| Frailty | 9 | 53.0 | 169.7 (88.3, 326.1) | 2.08 (0.91, 4.73) | 0.081 | 0.97 (0.33, 2.86) | 0.963 |
| Oral health Problem |  |  |  |  |  |  |  |
| No | 33 | 449.7 | 73.4 (52.2, 103.2) | 1 (Reference) | NA |  |  |
| Yes | 12 | 106.7 | 112.5 (63.9, 198.1) | 1.51 (0.78, 2.92) | 0.222 |  |  |
| Incontinence |  |  |  |  |  |  |  |
| No | 30 | 436.7 | 68.7 (48.0, 98.3) | 1 (Reference) | NA | 1 (Reference) | NA |
| Yes | 15 | 119.8 | 125.3 (75.6, 207.8) | 2.10 (1.12, 3.91) | 0.02 | 1.21 (0.58, 2.52) | 0.618 |
| Perceived health |  |  |  |  |  |  |  |
| Excellent/very good/good | 8 | 148.6 | 53.8 (26.9, 107.6) | 1 (Reference) | NA |  |  |
| Fair | 33 | 343.7 | 96.0 (68.2, 135.0) | 1.60 (0.74, 3.45) | 0.236 |  |  |
| Poor | 4 | 64.1 | 62.4 (23.4, 166.3) | 1.11 (0.33, 3.68) | 0.868 |  |  |
| Hypertension (measured) |  |  |  |  |  |  |  |
| No | 30 | 354.5 | 84.6 (59.2, 121.0) | 1 (Reference) | NA |  |  |
| Yes | 15 | 199.7 | 75.1 (45.3, 124.6) | 0.75 (0.40, 1.41) | 0.372 |  |  |
| Hypertension (diagnosed) |  |  |  |  |  |  |  |
| No | 8 | 171.4 | 46.7 (23.3, 93.3) | 1 (Reference) | NA | 1 (Reference) | NA |
| Yes | 37 | 385.1 | 96.1 (69.6, 132.6) | 1.99 (0.92, 4.26) | 0.079 | 1.24 (0.54, 2.84) | 0.612 |
| Dyslipidemia (diagnosed) |  |  |  |  |  |  |  |
| No | 20 | 299.5 | 66.8 (43.1, 103.5) | 1 (Reference) | NA |  |  |
| Yes | 25 | 256.9 | 97.3 (65.8, 144.0) | 1.58 (0.88, 2.84) | 0.128 |  |  |
| Diabetes mellitus (diagnosed) | |  |  |  |  |  |  |
| No | 27 | 397.7 | 67.9 (46.6, 99.0) | 1 (Reference) | NA | 1 (Reference) | NA |
| Yes | 18 | 158.8 | 113.4 (71.4, 180.0) | 1.76 (0.97, 3.20) | 0.064 | 0.92 (0.24, 3.61) | 0.909 |
| Cardiovascular Disease (diagnosed) | |  |  |  |  |  |  |
| No | 36 | 469.4 | 76.7 (55.3, 106.3) | 1 (Reference) | NA |  |  |
| Yes | 9 | 87.1 | 103.4 (53.8, 198.7) | 1.52 (0.73, 3.17) | 0.261 |  |  |
| Regular medication use |  |  |  |  |  |  |  |
| <5 | 27 | 414 | 65.2 (44.7, 95.1) | 1 (Reference) | NA | 1 (Reference) | NA |
| >=5 | 18 | 142.4 | 126.4 (79.6, 200.6) | 2.06 (1.13, 3.74) | 0.018 | 0.95 (0.41, 2.15) | 0.895 |
| Antihypertensive drugs use |  |  |  |  |  |  |  |
| No | 11 | 190.3 | 57.8 (32.0, 104.4) | 1 (Reference) | NA |  |  |
| Yes | 34 | 366.2 | 92.9 (66.3, 129.9) | 1.58 (0.80, 3.12) | 0.189 |  |  |
| Cardiovascular drugs use |  |  |  |  |  |  |  |
| No | 36 | 482.3 | 74.6 (53.8, 103.5) | 1 (Reference) | NA |  |  |
| Yes | 9 | 74.2 | 121.3 (63.1, 233.2) | 1.82 (0.88, 3.78) | 0.109 |  |  |
| Antidiabetics use |  |  |  |  |  |  |  |
| No | 28 | 421.1 | 66.5 (45.9, 96.3) | 1 (Reference) | NA | 1 (Reference) | NA |
| Yes | 17 | 135.3 | 125.6 (78.1, 202.1) | 2.05 (1.12, 3.75) | 0.02 | 2.04 (0.50, 8.31) | 0.319 |
| Anti-lipid drugs use |  |  |  |  |  |  |  |
| No | 22 | 331.5 | 66.4 (43.7, 100.8) | 1 (Reference) | NA |  |  |
| Yes | 23 | 224.9 | 102.3 (68.0, 153.9) | 1.51 (0.84, 2.71) | 0.167 |  |  |
| Antipsychotics use |  |  |  |  |  |  |  |
| No | 42 | 501.7 | 83.7 (61.9, 113.3) | 1 (Reference) | NA |  |  |
| Yes | 3 | 54.7 | 54.8 (17.7, 170.0) | 0.62 (0.19, 2.02) | 0.427 |  |  |
| Analgesics use |  |  |  |  |  |  |  |
| No | 36 | 498.4 | 72.2 (52.1, 100.1) | 1 (Reference) | NA | 1 (Reference) | NA |
| Yes | 9 | 58.1 | 155.0 (80.7, 297.9) | 2.04 (0.98, 4.25) | 0.055 | 1.40 (0.55, 3.53) | 0.479 |

BMI: bod mass index; CI: confidence interval; CSSA: comprehensive social security assistance; GAD: Generalised Anxiety Disorder; HR: hazard ratio; MCI: mild cognitive impairment; NA: not available; PHQ: Patient Health Questionnaire.

Supplementary Table S2 Factors associated with the risk of MCI among 660 participants after multiple imputation

| Variable | Crude HR (95%CI) | P Value | Multivariable-Adjusted HR (95%CI) | P Value |
| --- | --- | --- | --- | --- |
| Age |  |  |  |  |
| 60-69 | 1 (Reference) | NA | 1 (Reference) | NA |
| 70-79 | 4.00 (2.04, 7.83) | <0.001 | 2.99 (1.21, 7.36) | 0.017 |
| 80+ | 4.72 (1.88, 11.87) | 0.001 | 2.31 (0.75, 7.13) | 0.145 |
| Gender |  |  |  |  |
| Male | 1 (Reference) | NA |  |  |
| Female | 1.26 (0.65, 2.45) | 0.489 |  |  |
| BMI |  |  |  |  |
| Normal BMI | 1 (Reference) | NA |  |  |
| Abnormal BMI | 0.87 (0.48, 1.59) | 0.66 |  |  |
| Education |  |  |  |  |
| >6y | 1 (Reference) | NA | 1 (Reference) | NA |
| <=6y | 2.28 (1.24, 4.18) | 0.007 | 1.69 (0.89, 3.20) | 0.11 |
| Marriage |  |  |  |  |
| Married | 1 (Reference) | NA | 1 (Reference) | NA |
| Single/divorced/  separated/widowed | 2.81 (1.56, 5.04) | 0.001 | 2.06 (1.10, 3.88) | 0.025 |
| Live alone |  |  |  |  |
| No | 1 (Reference) | NA |  |  |
| Yes | 1.69 (0.78, 3.62) | 0.181 |  |  |
| Working status |  |  |  |  |
| Unemployed | 1 (Reference) | NA |  |  |
| Employed | 0.73 (0.26, 2.06) | 0.557 |  |  |
| Social assistance |  |  |  |  |
| No | 1 (Reference) | NA | 1 (Reference) | NA |
| Yes | 3.05 (1.57, 5.91) | 0.001 | 1.06 (0.43, 2.59) | 0.905 |
| CSSA |  |  |  |  |
| No | 1 (Reference) | NA |  |  |
| Yes | 1.95 (0.76, 4.98) | 0.166 |  |  |
| Social media use |  |  |  |  |
| No | 1 (Reference) | NA | 1 (Reference) | NA |
| Yes | 0.40 (0.22, 0.73) | 0.003 | 0.71 (0.37, 1.39) | 0.318 |
| Depression (PHQ9) |  |  |  |  |
| No | 1 (Reference) | NA |  |  |
| Yes | 0.58 (0.24, 1.38) | 0.22 |  |  |
| Anxiety (GAD7) |  |  |  |  |
| No | 1 (Reference) | NA |  |  |
| Yes | 0.52 (0.21, 1.33) | 0.174 |  |  |
| Loneliness |  |  |  |  |
| Total score =0 | 1 (Reference) | NA |  |  |
| Total score >=1 | 0.67 (0.37, 1.21) | 0.188 |  |  |
| Insomnia |  |  |  |  |
| No | 1 (Reference) | NA |  |  |
| Yes | 1.39 (0.77, 2.50) | 0.274 |  |  |
| Sarcopenia |  |  |  |  |
| No | 1 (Reference) | NA |  |  |
| Yes | 2.01 (0.85, 4.75) | 0.113 |  |  |
| Drinker |  |  |  |  |
| No | 1 (Reference) | NA |  |  |
| Yes | 1.61 (0.50, 5.21) | 0.428 |  |  |
| Smoker |  |  |  |  |
| Non-smoker | 1 (Reference) | NA |  |  |
| Current smoker | 1.42 (0.34, 5.90) | 0.626 |  |  |
| Ex-smoker | 0.67 (0.24, 1.88) | 0.45 |  |  |
| Frailty |  |  |  |  |
| No frailty | 1 (Reference) | NA | 1 (Reference) | NA |
| Pre-frailty | 1.10 (0.57, 2.12) | 0.784 | 1.11 (0.56, 2.22) | 0.759 |
| Frailty | 2.19 (0.96, 4.98) | 0.061 | 1.21 (0.47, 3.09) | 0.69 |
| Oral health Problem |  |  |  |  |
| No | 1 (Reference) | NA |  |  |
| Yes | 1.28 (0.66, 2.49) | 0.458 |  |  |
| Incontinence |  |  |  |  |
| No | 1 (Reference) | NA | 1 (Reference) | NA |
| Yes | 2.02 (1.09, 3.76) | 0.026 | 1.32 (0.68, 2.59) | 0.412 |
| Perceived health |  |  |  |  |
| Excellent/very good/good | 1 (Reference) | NA |  |  |
| Fair | 1.71 (0.79, 3.69) | 0.176 |  |  |
| Poor | 1.19 (0.36, 2.95) | 0.776 |  |  |
| Hypertension (measured) |  |  |  |  |
| No | 1 (Reference) | NA |  |  |
| Yes | 0.78 (0.41, 1.46) | 0.429 |  |  |
| Hypertension (diagnosed) |  |  |  |  |
| No | 1 (Reference) | NA |  |  |
| Yes | 1.75 (0.82, 3.76) | 0.151 |  |  |
| Dyslipidemia (diagnosed) |  |  |  |  |
| No | 1 (Reference) | NA |  |  |
| Yes | 1.48 (0.82, 2.66) | 0.194 |  |  |
| Diabetes mellitus (diagnosed) |  |  |  |  |
| No | 1 (Reference) | NA |  |  |
| Yes | 1.63 (0.90, 2.97) | 0.107 |  |  |
| Cardiovascular Disease (diagnosed) | |  |  |  |
| No | 1 (Reference) | NA |  |  |
| Yes | 1.48 (0.71, 3.08) | 0.294 |  |  |
| Regular medication use |  |  |  |  |
| <5 | 1 (Reference) | NA | 1 (Reference) | NA |
| >=5 | 1.85 (1.02, 3.35) | 0.044 | 0.89 (0.39, 2.00) | 0.755 |
| Antihypertensive drugs use |  |  |  |  |
| No | 1 (Reference) | NA |  |  |
| Yes | 1.36 (0.69, 2.68) | 0.378 |  |  |
| Cardiovascular drugs use |  |  |  |  |
| No | 1 (Reference) | NA |  |  |
| Yes | 1.65 (0.79, 3.43) | 0.18 |  |  |
| Antidiabetics use |  |  |  |  |
| No | 1 (Reference) | NA | 1 (Reference) | NA |
| Yes | 1.81 (0.99, 3.30) | 0.054 | 1.76 (0.85, 3.64) | 0.125 |
| Anti-lipid drugs use |  |  |  |  |
| No | 1 (Reference) | NA |  |  |
| Yes | 1.44 (0.80, 2.59) | 0.219 |  |  |
| Antipsychotics use |  |  |  |  |
| No | 1 (Reference) | NA |  |  |
| Yes | 0.65 (0.20, 2.11) | 0.475 |  |  |
| Analgesics use |  |  |  |  |
| No | 1 (Reference) | NA | 1 (Reference) | NA |
| Yes | 1.93 (0.93, 4.00) | 0.078 | 1.61 (0.68, 3.85) | 0.28 |

BMI: bod mass index; CI: confidence interval; CSSA: comprehensive social security assistance; GAD: Generalised Anxiety Disorder; HR: hazard ratio; MCI: mild cognitive impairment; NA: not available; PHQ: Patient Health Questionnaire.
